# Evaluation of transient respiratory motion artifact at gadoxetate disodium-enhanced MRI—Influence of different contrast agent application protocols

**DOI:** 10.1371/journal.pone.0200887

**Published:** 2018-07-19

**Authors:** Kristina I. Ringe, Christian von Falck, Hans-Jürgen Raatschen, Frank Wacker, Jan Hinrichs

**Affiliations:** Hannover Medical School, Department of Diagnostic and Interventional Radiology, Hannover, Germany; Johns Hopkins School of Medicine, UNITED STATES

## Abstract

**Purpose:**

To evaluate transient severe respiratory motion artifacts (TSM) at gadoxetate disodium-enhanced MRI dependent on the mode of contrast agent application.

**Methods:**

200 patients (71f, 129m; mean 51y) were included in this retrospective IRB-approved study. Contrast application protocols (n = 4) differed with regards to injection rate (2ml or 1ml/sec), dose (weight-based or fixed 10ml) and supplemental oxygen administration (yes/no). SNR measurements were performed in the aorta and portal vein. Qualitatively, three readers assessed arterial phase image quality and TSM independently (4- and 5-point scale, respectively). Quantitative and qualitative results were compared (Kruskal-Wallis test, Dunn’s multiple comparison test). The influence of different contrast agent application parameters on the occurrence of respiratory motion artifacts was assessed (univariate analysis). Interrater agreement and reliability were calculated (intraclass correlation coefficient, ICC)).

**Results:**

Use of a lower contrast injection rate resulted in significantly higher arterial SNR in the aorta and portal vein (p<0.05). TSM was observed in 12% of examinations. Neither injection rate, contrast dose, nor oxygen had a significant influence. Interrater agreement and reliability for evaluation of image quality and respiratory motion were substantial/ almost perfect (ICC = 0.640–0.915).

**Conclusions:**

Technical factors regarding the specific mode of contrast application do not seem to significantly reduce the incidence of severe transient respiratory motion artifacts.

## Introduction

Gadoxetate disodium is a liver-specific contrast agent, which has been approved by the FDA in 2005 for lesion detection and characterization. In patients with normal liver and kidney function hepatic uptake and subsequent biliary excretion are in the range of approximately 50% [1]. The specific pharmacokinetic and pharmacodynamic properties of this contrast agent allow for acquisition of a contrast dynamic and additional hepatocyte phase imaging. In this context, proper dynamic phase imaging is fundamental for lesion detection and characterization in the healthy as well as in the cirrhotic liver [2,3].

**Fig 1:**
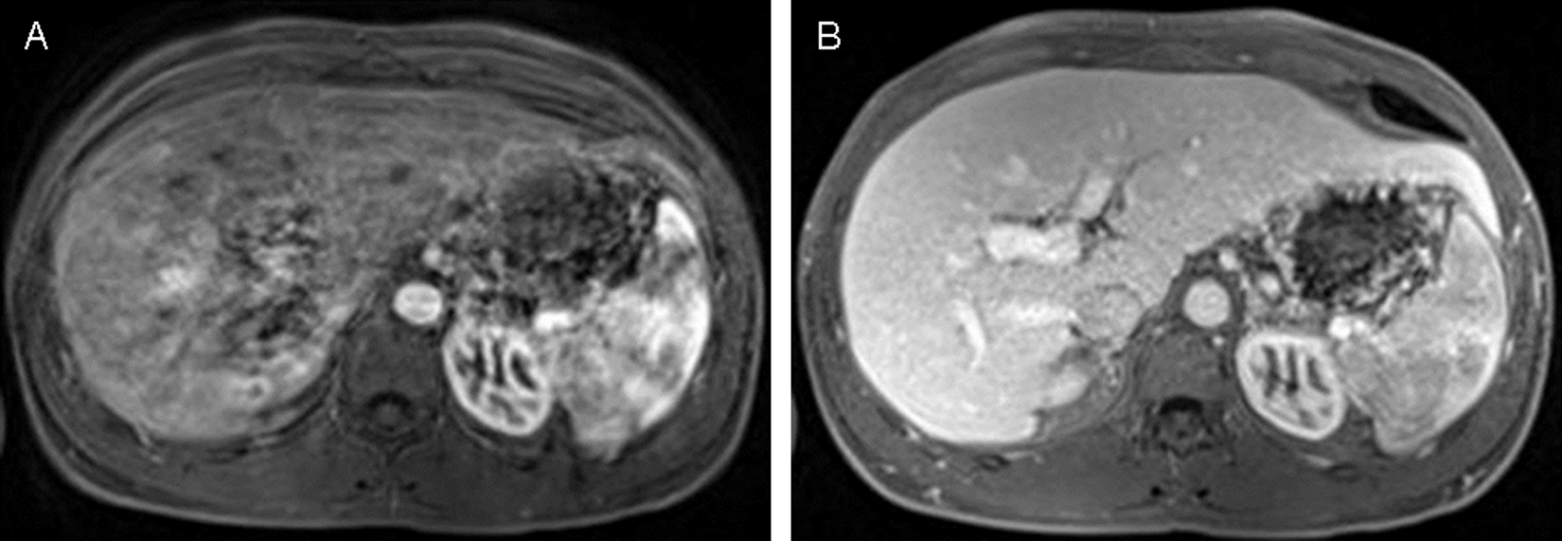
27 year old male patient with diagnosis of primary sclerosing cholangitis. Gadoxetate disodium-enhanced liver MRI in the arterial (A) and portalvenous phase (B). Self-limiting severe transient motion can be appreciated in the arterial phase, causing significant degradation of image quality, which is resolved in the portalvenous phase.

Increasingly, an association has been described between the injection of gadoxetate disodium and respiratory motion artifacts in the arterial phase of the contrast dynamic. This phenomenon, also referred to as TSM (transient severe motion), is typically self-limiting, lasts for about 10 to 20 seconds, and may be accompanied by the patients’ subjective feeling of the inability to catch breath [4,5]. In this context the term “severe” implies a significant degradation of image quality, which has in most previous studies been assessed subjectively on a five-point scale with a score of at least 4, and has been shown to have high interrater agreement [6,7]. Even though this episode is only temporarily, it may have destructive effects on arterial phase MRI, in the worst-case rendering images non-diagnostic (Fig 1).

The incidence of transient severe respiratory motion artifacts throughout the literature is not consistent with a reported range between 2.4 and up to 18% [4,8,9]. Furthermore, there seems to be a distinct regional difference between studies from the USA and those from Europe or Asia. However, the exact pathophysiology of this phenomenon is still unknown [10]. Several patient-related risk factors are being discussed including a possible non-allergic mechanism [6], an underlying chronic obstructive pulmonary disease (COPD) [6] or known allergy to iodinated contrast agent [10], as well as a prior episode of TSM after contrast injection [7,11]. Furthermore, MR specific risk factors are under debate, most importantly the total injected contrast volume [6].

In order to elucidate this phenomenon, the purpose of our study was to evaluate the incidence of severe transient respiratory motion artifacts in clinical routine at gadoxetate disodium-enhanced MRI dependent on the mode of contrast agent application, specifically by variation of contrast dose, injection rate and supplemental nasal oxygen application.

## Materials and methods

### Patients

This retrospective study was approved by the IRB of Hannover Medical School with a waiver of consent granted, and the investigation has been conducted according to the principles expressed in the Declaration of Helsinki. From October 2012 to February 2015, 200 patients (129 males, 71 females; mean age 51 years) referred for gadoxetate disodium-enhanced MRI in clinical routine were included in this study. Inclusion criteria were as follows: completion of the entire MRI scan; administration of gadoxetate disodium; patient age of at least 18 years. Patient characteristics are provided in detail in Table 1.

**Table 1:** Contrast agent application protocols (group 1–4) for acquisition of gadoxetate disodium-enhanced MR images and characteristics of patients included in each group.

Protocol No.	Injection rate	Contrast dose	BMI	Nasal oxygen	Number of patients	Sex	Age	MRI indication
**1**	2 ml/sec	0.025 mmol/kg (= 0.1ml/kg)	27 (16–42)	no	50	male = 40 female = 10	mean 52y (26-75y)	Cirrhosis (n = 12) PSC (n = 16) Tumor (n = 17) Other n = (5)
**2**	2 ml/sec	fixed 10 ml	24 (16–35)	no	50	male = 29 female = 21	mean 50y (19-84y)	Cirrhosis (n = 10) PSC (n = 23) Tumor (n = 11) Other (n = 6)
**3**	1 ml/sec	0.025 mmol/kg (= 0.1ml/kg)	27 (19–45)	no	50	male = 30 female = 20	mean 50y (19-73y)	Cirrhosis (n = 10) PSC (n = 11) Tumor (n = 20) Other (n = 9)
**4**	1ml /sec	0.025 mmol/kg (= 0.1ml/kg)	26 (16–46)	yes	50	male = 30 female = 20	mean 52y (18-79y)	Cirrhosis (n = 11) PSC (n = 11) Tumor (n = 19) Other (n = 9)

BMI = body mass index (mean, range in brackets); PSC = primary sclerosing cholangitis

### MR imaging

MR examinations were performed on a 1.5T system (Magnetom Avanto, Siemens Healthineers, Erlangen, Germany) using phased-array surface coils that covered the whole abdomen. All patients underwent a routine imaging protocol of the liver including axial T1-weighted VIBE (volumetric interpolated breath-hold examination) sequences in the arterial, portalvenous and delayed phase after the injection of gadoxetate disodium with a slice thickness of 2mm. Contrast-enhanced dynamic sequences were acquired in inspiration, starting 4 seconds after bolus detection in the thoracic descending aorta. The interscan delay between the arterial and portalvenous phase was one breathhold, and 60 seconds between the portalvenous and delayed phase, respectively. The acquisition time for each of these sequences was 16 seconds.

During the study period, the specific mode of contrast agent application differed with regard to injection rate (1 ml/sec or 2 ml/sec), contrast dose (0.025 mmol/kg body weight or fixed 10 ml) and a possible nasal oxygen application (2 l/min; yes or no) due to changes in clinical routine. This resulted in four different protocols (Table 1). For comparison of these different protocols, 50 consecutive patients were randomly chosen in whom images were acquired using these specific contrast agent application parameters, were chosen from our clinical database and included in each group.

### Image analysis

Image analysis was performed on a commercially available workstation (Visage 7.1; Pro Medicus Inc., Melbourne, VIC, Australia).

#### Quantitative analysis

For quantitative image analysis, two board-certified radiologists (both blinded to the mode of contrast agent application) in consensus performed signal-to-noise ratio (SNR) measurements in the arterial phase. Regions of interest (ROI; size 154mm^2^) were placed in the aorta at the level of the celiac trunk and in the main portal vein at the level of the liver hilum, respectively. Noise estimates were derived in each dataset outside the body in the vicinity of the liver. SNR was calculated as follows: mean signal of the vessel divided by the standard deviation of noise.

#### Qualitative image analysis

Qualitative image analysis was performed independently by three different board-certified radiologists, blinded to the mode of contrast agent application as well as to the findings of the other radiologists. Each radiologist had knowledge about the appearance of respiratory motion artifact and the differentiation from other sources of image degradation, e.g. truncation. Arterial phase image quality was evaluated by means of a four-point scale [5]. Score 1: no contrast material in the hepatic artery; Score 2: early arterial phase with contrast material in the hepatic artery but not in the portal vein; Score 3: adequate late arterial with portal vein or early parenchymal enhancement; Score 4: too late with strong parenchymal or hepatic venous enhancement. Further, motion-related artifacts were assessed in each phase of the contrast dynamic as well as in the pre-contrast scan using a five-point scale, which has been shown to have a high interrater reliability [5]. Score 1: no motion-related artifact; Score 2: minimal motion-related artifact with no effect on diagnostic quality; Score 3: moderate motion-related artifact with some, but not severe effect on diagnostic quality; Score 4: severe motion-related artifact, but images are still interpretable; Score 5: extensive motion-related artifact resulting in non-diagnostic image quality. The occurrence of TSM artifact was defined similar to previous studies: a mean motion score on non-enhanced images ≤ 2, in combination with a mean motion score on arterial phase images ≥ 4 and a mean motion score on portalvenous phase images ≤ 2 [6,7].

### Statistical analysis

Statistical analysis was performed using SPSS (version 22; IBM Corporation, New York, USA) and GraphPad Prism software (version 7, GraphPad Software, Inc., USA). To test for potential differences in sex distribution and underlying disease between different protocol groups a Chi^2^-test was performed; to test for potential differences regarding age distribution an ANOVA was performed (after testing for a Gaussian and equal distribution using the D’Agostino and Pearson normality test). SNR, arterial phase image quality and motion scores between different protocols were compared using a Kruskal-Wallis test and Dunn’s multiple comparison test, respectively. A post-hoc power analysis was performed using G*Power 3.1 (Heinrich-Heine University, Düsseldorf). Assuming an ɑ-error of 0.05, an effective size f of 0.4079, and sample size of 200, the calculated power is 0.999. The incidence of TSM artifact was calculated for each contrast agent application protocol. Further, the rate of TSM artifact using different contrast agent application parameters was evaluated by means of univariate analysis. In addition, interrater agreement and interrater reliability for grading of arterial phase image quality and motion-related artifact was calculated using the intraclass correlation coefficient (ICC) according to McGraw and Wong [12], applying a two-way mixed model. While agreement was defined as the degree to which ratings given by different judges (here: assigned motion artifact scores by different readers) are identical, reliability refers to the consistency of ratings and the extent of variability [13]. ICC was interpreted as follows: a value less than 0.20 indicated poor agreement; a value of 0.21–0.40, fair agreement; a value of 0.41–0.60, moderate agreement; a value of 0.61–0.80, substantial agreement; and a value of 0.81–1.00, almost perfect agreement [14]. For all measurements p<0.05 indicated a significant difference.

## Results

### Patients

Even though there were more males in protocol group 1, and more patients with PSC in protocol group 2, respectively, overall there was no statistical difference between all groups with regard to age and sex distribution as well as underlying disease (p>0.05). The mean contrast dose in protocol group 2 was 0.14 ml / kg body weight (range 0.1–0.2 ml / kg body weight), as opposed to 0.1 ml / kg body weight in all other groups.

### Quantitative results

**Fig 2:**
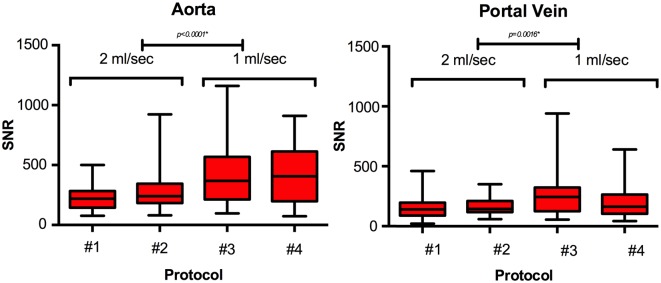
Arterial phase SNR in the aorta (left) and in the portal vein (right), comparison of different contrast agent application protocols (#1–4). SNR in the aorta and portal vein were significantly higher in protocol groups using the slower injection rate of 1 ml/sec, as compared to an injection rate of 2 ml/sec (p<0.0001 and p = 0.0016). * indicates a statistical significance.

Arterial phase SNR in the aorta and portal vein were significantly higher in protocol groups using the slower injection rate of 1 ml/sec, as compared to an injection rate of 2 ml/sec (p<0.0001 and p = 0.0016). Mean aortal SNRs were 224 (group 1), 284 (group 2), 407 (group 3) and 437 (group 4); mean SNRs in the portal vein were 154 (group 1), 164 (group 2), 249 (group 3) and 196 (group 4), respectively (Fig 2). Looking at the groups with an injection rate of 2 ml/sec only, there was no significant difference regarding SNR in the aorta and portal vein between patients in whom the contrast agent was administered based on weight (group 1) and those who received a fixed bolus of 10 ml (group 2; p>0.05).

### Qualitative results

Arterial phase image quality, in terms of timing of arterial phase image acquisition was comparable between different contrast agent application protocols with a mean score of 2.8 (group 1), 2.8 (group 2), 2.7 (group 3) and 2.5 (group 4), respectively. Interrater agreement and reliability for assessment of arterial phase image quality were substantial (both ICC = 0.80 and p<0.001; 95% confidence intervals 0.743–0.841 and 0.745–0.842, respectively).

**Fig 3:**
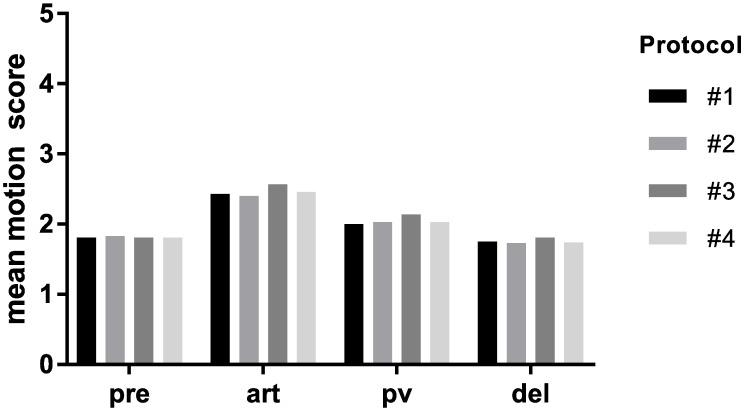
Mean motion scores in different phases of the contrast dynamic after injection of gadoxetate disodium, comparison of different contrast agent application protocols (#1–4). Mean motion score as assessed on a 5-point scale by three readers, in the pre-contrast (pre), arterial (art), portalvenous (pv) and delayed (del) phase, respectively.

**Fig 4:**
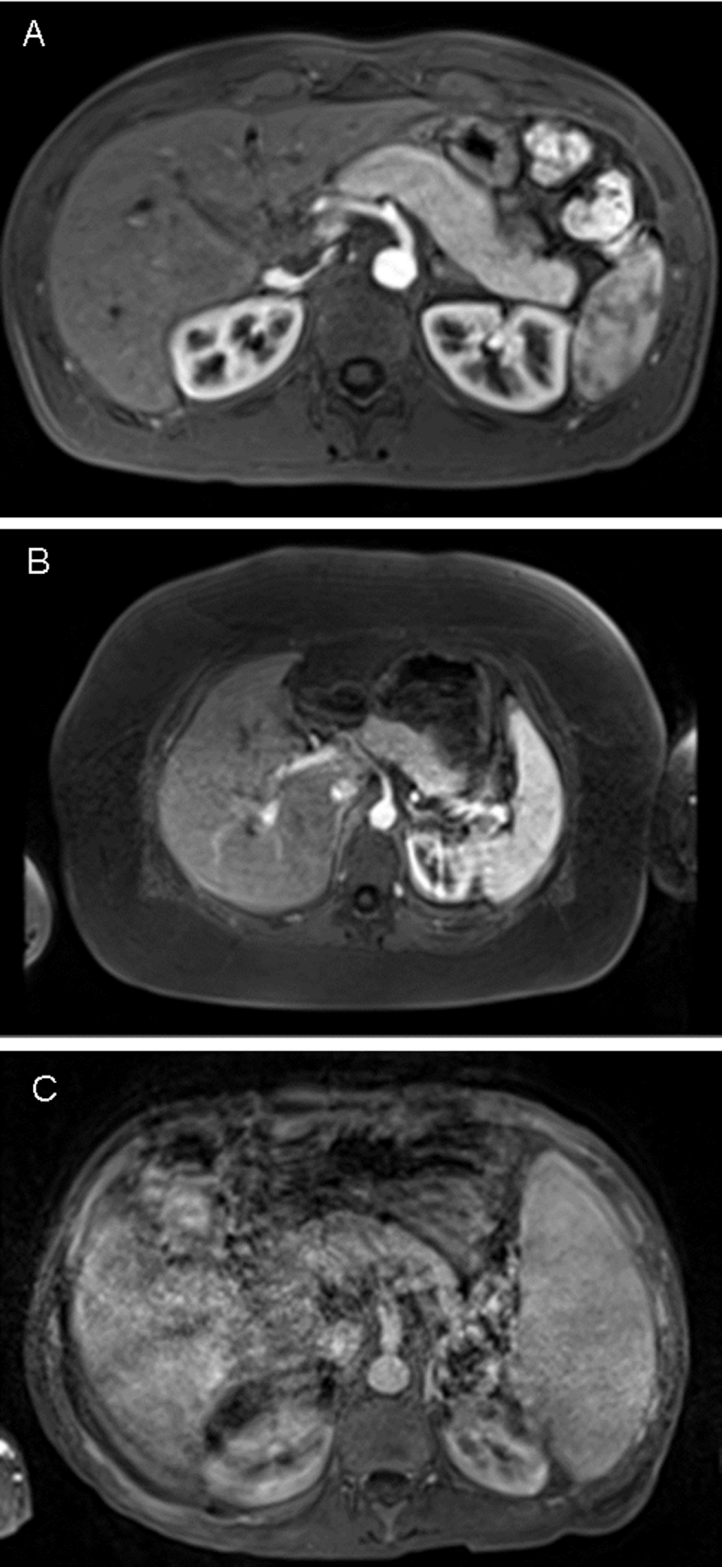
Grading of motion-related artifacts after gadoxetate disodium injection on a 5-point scale, exemplarily arterial phase datasets. A: Rated as grade 1 by all three readers; B: rated as grade 3 by all three readers; C: rated as grade 5 by all three readers.

Regarding the scoring of motion artifact after contrast injection, there was no significant difference between contrast application protocols as assessed by all three readers and as assessed in all phases of the contrast dynamic (p>0.05 for all phases of the contrast dynamic). Mean motion scores were lowest in the pre-contrast scan (1.6–1.8), and highest in the arterial phase (2.4–2.6), independent of the contrast application protocol applied (Fig 3). Interrater agreement and reliability for evaluation of motion artifact in different phases of the contrast dynamic were substantial to almost perfect; pre-contrast phase: ICC = 0.702 (0.360–0.837) and 0.818 (0.769–0.858); arterial phase: ICC = 0.861 (0.669–0.927) and 0.915 (0.892–0.933); portalvenous phase: ICC = 0.821 (0.464–0.917) and 0.912 (0.889–0.931); delayed phase: ICC = 0.640 (0.206–0.810) and 0.801 (0.748–0.845) with p<0.0001 for all calculations (Fig 4).

Severe transient motion artifact was observed in 24 patients, summing up to an overall incidence of 12%. Looking in detail at the different contrast agent application protocols, severe motion artifact was observed in n = 5 (= 10%; group 1), n = 7 (= 14%; group 2), n = 4 (= 8%; group 3) and n = 7 (14%; group 4) patients, respectively. At univariate analysis, neither injection rate (p = 1.0), nor contrast dose (p = 0.617), nor additional oxygen administration (p = 0.316) had a significant influence on the rate of severe transient motion at gadoxetate disodium-enhanced MRI. Out of the 24 studies with severe transient motion artifact, the arterial phase was rated as non-diagnostic in 5 cases. Of note, in three of these cases the fixed 10ml contrast dose was applied (protocol number 2); in one case each protocol 1 and 4 were applied.

## Discussion

In this study, we evaluated the occurrence of transient respiratory motion artifact in clinical routine at gadoxetate disodium-enhanced MRI dependent on the mode of contrast agent application. The overall incidence of severe motion artifact in our study population was 12%. Based on our results, technique specific factors regarding contrast administration (injection rate, volume, supplemental oxygen application) did not seem to significantly reduce the incidence of severe motion. These findings are important as severe motion artifacts at gadoxetate disodium-enhanced MRI constitute an ongoing problem in clinical routine.

Different strategies have been suggested how to minimize the occurrence of severe transient motion artifacts and how to cope with this still unaccounted for phenomenon. These approaches include the reduction of acquisition time and obtaining more than one arterial phase (e.g. “triple-arterial” phase) [5], the use of short-breath-hold techniques [15], the reduction of contrast dose, the use of a modified breathing command [16], or even the use of an alternative contrast agent [17].

Results regarding the use of a lower contrast dose are not consistent throughout previous studies. Davenport et al initially described in 2013 that patients reporting subjective dyspnea had a significantly lower body weight [17]. However, in a matched within patient cohort study published by the same group neither dose by weight nor dose by volume were significant predictors of severe motion artifacts [4]. This was substantiated in a dose-toxicity relationship study shortly afterwards, which indicated that not patient weight but rather the injected contrast volume has an independent role [6]. Furthermore, Hayashi et al evaluated possible risk factors associated with TSM in patients who received a weight-based dose of 0.025 mmol/kg. They concluded, that severe motion artifacts were observed significantly more often in patients with a higher body weight (62.5 kg ± 14.0 vs. 66.6 kg ± 12.6; p = 0.03), again supporting the idea that TSM is dose-related [18]. In our present study, the use of a weight-based contrast dose (0.1ml / kg) as opposed to a fixed dose of 10 ml (resulting in a range of 0.1–0.2 ml/kg) did not have a significant influence on the rate of severe motion artifact.

Even though the change of contrast injection rate (2 ml / sec vs. 1 ml / sec) did not result in a substantial reduction of TSM in our study, overall arterial phase image quality (as assessed quantitatively by SNR measurements) was significantly better with the lower injection rate, a finding that is in line with previous reports [19]. As the standard dosing of gadoxetate disodium (0.025 mmol / kg body weight) is rather low as compared to other contrast agents (mostly 1 mmol /kg body weight), the contrast bolus may be prolonged by using a lower injection rate. This results in a more favorable bolus configuration [20,21].

The rationale for giving patients oxygen via a nasal tube during the MRI was based on our observation of patients who had trouble holding their breath for a certain amount of time. Although this was independent from the contrast agent being used, we hypothesized that the occurrence of severe motion artifacts could be reduced by giving oxygen. However, despite oxygen administration severe motion artifacts were still observed in 7 patients (14%). One possible explanation for the lack of a positive effect of oxygen on the rate of TSM could be that the timing of arterial phase image acquisition in this patient group was slightly earlier as compared to the other protocol groups. However, at this point we can only speculate that slightly later image acquisition in combination with oxygen would have resulted in a significant reduction of severe artifacts. To the best of our knowledge, there is no study in which oxygen was administered with the specific aim of reducing severe respiratory motion artifacts at gadoxetate disodium-enhanced MRI. Our results substantiate the findings of previous studies in which oxygen saturation [18,22] or respiratory waveforms [9] were monitored during contrast-enhanced imaging. Hayashi et al for example observed no significant change in oxygen saturation after gadoxetate disodium-injection (dose: 0.025 mmol/ kg administered at a rate of 1 ml/sec) [18]. Similarly, Motosugi et al found that severe motion artifacts in the arterial phase were associated with breath-hold failure but not with subjective feelings of dyspnea or substantial decrease of SpO_2_ [22]. McClellan et al only recently demonstrated that maximal hepatic arterial phase breath-holding duration was reduced after gadoxetate disodium injection (as compared to normal saline and gadoterate dimeglumine) in healthy volunteers, associated with motion artifacts [23]. Further, it was shown in a rat model, that arterial blood gases are not affected by the injection of gadoxetate disodium [24]. Looking at the results of our study and those of previous reports in synopsis, oxygen deficiency does not seem to cause TSM.

Our study does have certain limitations. Due to the retrospective design of the study we were not able to evaluate the occurrence of self-reported dyspnea during contrast injection, which is known to have a certain association with severe respiratory motion artifact, even though these two incidents are not ultimately linked [22]. Also, even though the study population overall comprised 200 patients, only 50 patients were included in each protocol group in order to keep group sizes and patient characteristics homogeneous and comparable.

In conclusion, severe transient respiratory motion artifacts are an only recently addressed phenomenon at gadoxetate disodium-enhanced MRI, known to cause significant degradation of arterial phase image quality. The overall incidence of severe motion artifacts in our study was 12%, which is in line with previous reports. However, neither variation of contrast dose, injection rate, nor the supplemental nasal administration of oxygen seems to significantly reduce the incidence of this artifact in clinical routine. From our results, a weight-based contrast dose administered at a flow of 1 ml/ sec seems to be the most favorable approach in clinical routine at this time due to the highest SNR as assessed in the aorta and portal vein, whereas a potential benefit of oxygen could not demonstrated.
